# Integrating multi-isotope calibration and infrared-assisted digestion for robust and sustainable multielemental determination in agroalimentary matrices by ICP-MS

**DOI:** 10.3389/fchem.2026.1799245

**Published:** 2026-06-15

**Authors:** Florencia Cora Jofre, Pablo Hugo Pacheco, Marianela Savio

**Affiliations:** 1 Facultad Ciencias Exactas y Naturales, Universidad Nacional de La Pampa, Santa Rosa, La Pampa, Argentina; 2 Instituto de Ciencias de la Tierra y Ambientales de La Pampa, Consejo Nacional de Investigaciones Científicas y Técnicas, Universidad Nacional de La Pampa (INCITAP-CONICET-UNLPam) (INCITAP), Santa Rosa, La Pampa, Argentina; 3 Instituto de Química de San Luis, Consejo Nacional de Investigaciones Científicas y Técnicas-Universidad Nacional de San Luis, Facultad de Química, San Luis, Argentina

**Keywords:** agroalimentary matrices, ICP-MS, infrared-assisted digestion, multielemental analysis, multi-isotope calibration, sustainable analytical chemistry

## Abstract

Multi-isotope calibration (MICal) was evaluated as an alternative quantitative strategy for multi-elemental determination by inductively coupled plasma mass spectrometry (ICP-MS) in complex agroalimentary matrices. Infrared-assisted digestion (IRAD) was employed as a rapid and acid-efficient sample preparation approach. Method performance was assessed using certified reference material and further demonstrated through the analysis of real samples, including compound feeds and animal-derived matrices. MICal showed well-defined linear behavior based on paired digest + standard and digest + blank solutions, enabling intrinsic matrix matching and the identification of isotope-specific spectral and matrix-related interferences. Accurate and precise quantification was achieved for all selected isotopes, with recoveries ranging from 85% to 118% and relative standard deviations (RSD) below 5%. Limits of quantification (LOQ) were suitable for both nutritional assessment and contaminant monitoring, ranging from 0.02 mg kg^-1^ for Mo to 47 mg kg^-1^ for Zn. Sustainability aspects were evaluated using complementary green and white analytical chemistry approaches. IRAD significantly reduced reagent consumption and energy demand, while the combined digestion–calibration workflow provided a balanced compromise between analytical performance, environmental impact, and operational efficiency. The method was successfully applied to the determination of Cd, Cu, Mo, Pb, V, and Zn in real samples, with concentrations evaluated against dietary recommendations and tolerance limits established by the National Research Council (NRC). Toxic elements such as Cd and Pb were below detection limits (LOD) in all samples. Overall, the results demonstrate that integrating MICal with IRAD provides a robust, sustainable, and practically efficient strategy for routine multielemental analysis of agroalimentary matrices.

## 1 Introduction

Reliable multielemental analysis of agroalimentary products is essential for nutritional evaluation, food safety control, and environmental monitoring. Inductively coupled plasma mass spectrometry (ICP-MS) is widely recognized for its high sensitivity and multielement capability; however, its quantitative performance strongly depends on the calibration strategy, particularly when analyzing complex matrices (El Hosry et al., 2023; Huang and Li, 2025; Mazarakioti et al., 2022; Nguyen et al., 2023). Ideally, standards and samples should share identical physicochemical environments; however, this is rarely achievable in practical agri-food analysis. Agroalimentary samples typically contain high levels of organic matter, proteins, carbohydrates, lipids, salts, and easily ionizable elements, which can alter plasma conditions and ionization efficiency, leading to significant matrix-induced signal suppression or enhancement, especially when mild or partial digestion strategies are applied and residual matrix components reach the plasma (de Mello et al., 2022; Ibourki et al., 2023; Sheehan and Furey, 2024; Soares et al., 2023).

Traditional external calibration (EC) is simple and widely applied, but it often fails to compensate for matrix effects when standards and samples do not share identical characteristics. Standard addition (SA) method is widely recognized as an effective strategy for compensating matrix effects. However, its application requires the preparation of multiple calibration solutions for each individual sample, resulting in higher reagent consumption, longer analysis time, and reduced analytical throughput. Consequently, there is a growing need for fast, robust, and matrix-adapted calibration strategies capable of ensuring accurate quantification across heterogeneous agroalimentary matrices (Carter et al., 2018; Donati and Amais, 2019; Virgilio et al., 2018).

**FIGURE 1:**
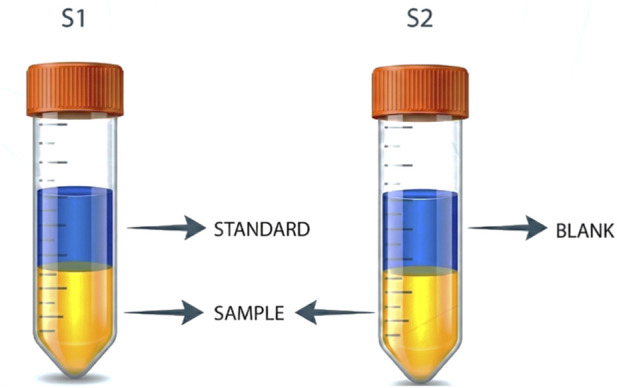
MICal strategy schematic illustration applied to ICP-MS analysis.

In recent years, multi-signal calibration approaches have been proposed as an alternative to classical signal–concentration relationships. These strategies construct calibration functions using instrumental signals on both axes, enabling accurate quantification with a reduced number of calibration solutions (Maringolo et al., 2024; Virgilio et al., 2017; 2018). Within this framework, Multi-Isotope Calibration (MICal), introduced by Virgilio et al. (2018), exploits the signals of multiple naturally occurring isotopes of an analyte measured in two solutions containing identical sample amounts: one prepared by mixing the sample digest with a standard solution (S1) and the other by mixing the sample digest with an analytical blank (S2) (Figure 1). Because both solutions share the same matrix composition, MICal inherently provides matrix matching while maintaining a simple and rapid workflow (de Higuera et al., 2020; Virgilio et al., 2018).

Since its introduction, MICal has been explored in a variety of analytical contexts, demonstrating its potential as a matrix-adapted calibration strategy for ICP-MS. Previous studies have shown that MICal can deliver recoveries comparable to or better than EC and SA, while reducing calibration effort and solution consumption (Borges de Oliveira et al., 2025; de Higuera et al., 2020; Virgilio et al., 2018; Wheal and Wilkes, 2021; Zhou et al., 2022). These findings highlight its suitability for matrices prone to spectral and matrix-related interferences.

In parallel, increasing attention has been devoted to the development of faster and more sustainable sample preparation strategies for elemental analysis. Infrared-assisted digestion (IRAD) has emerged as an attractive alternative to conventional microwave-assisted or block digestion procedures. By using directional infrared radiation, IRAD enables rapid heating and efficient oxidation of organic matter, achieving effective sample mineralization with reduced acid consumption, shorter digestion times, and lower energy demand. These features make IRAD particularly suitable for agroalimentary and biological matrices, where high organic content and compositional heterogeneity pose analytical challenges (Cora Jofre and Savio, 2024).

Previous studies have demonstrated that IRAD can achieve complete or near-complete digestion of complex matrices while minimizing reagent consumption and analytical waste, in line with the principles of Green Analytical Chemistry (GAC) (Anastas and Warner, 1998; Gałuszka et al., 2013). In addition, the simplicity of IRAD instrumentation and its compatibility with routine laboratory workflows support its application in high-throughput elemental analysis (Nowak et al., 2021).

Despite the demonstrated potential of MICal and IRAD when considered separately, their combined application has not yet been systematically explored for the multielemental analysis of agroalimentary matrices intended for animal nutrition, such as compound feeds. These matrices remain particularly challenging due to their variable composition and high organic content, which can exacerbate matrix effects even after digestion.

In this context, the present study evaluates the integration of IRAD and MICal for the determination of Cd, Cu, Mo, Pb, V, and Zn in agroalimentary matrices by ICP-MS. Analytical performance was assessed in terms of linearity, limits of detection (LOD) and quantification (LOQ), precision, and accuracy. In addition, the analytical workflow was evaluated from both GAC and White Analytical Chemistry (WAC) perspectives, highlighting its potential as a robust, sustainable, and practically efficient alternative for routine multielemental analysis.

## 2 Materials and methods

### 2.1 Samples and reagents

All reagents were of analytical grade. Nitric acid (HNO_3_, 65% m m^−1^ Merck, Darmstadt, Germany) and hydrogen peroxide (H_2_O_2_, 30% m m^−1^ Sigma-Aldrich) were used for sample digestion. Ultrapure water (resistivity ≥18.2 MΩ cm) was used throughout the study. Monoelemental ICP-MS standard solutions of Cd, Cu, Mo, Pb, V, and Zn were employed for spiking and calibration. A diluted nitric acid solution (0.14 mol L^-1^ HNO_3_) was used as the blank matrix.

A total of 13 real samples were included to evaluate the applicability of the method to matrices of varying complexity. The analytical performance was assessed using representative agroalimentary matrices. A certified reference material (CRM) of bovine liver (RM-Agro E3001a) was used for method validation. Additional samples included pelleted compound feed and processed maize-based food products.

### 2.2 Infrared-assisted digestion (IRAD)

Sample digestion was performed using IRAD, following previously reported methodologies as a rapid and environmentally favorable alternative to conventional microwave-assisted digestion (MWAD). The digestion was carried out under controlled IR radiation using a laboratory-scale prototype operating at 500 W, reaching approximate temperatures of 190 °C, with a total digestion time of approximately 30 min for 18 samples. This approach has been shown to reduce acid consumption, digestion time, and energy demand, while providing clear digests suitable for elemental analysis of organic-rich matrices by ICP-MS (Cora Jofre et al., 2021; Cora Jofre and Savio, 2024).

Approximately 0.15 g of homogenized sample was accurately weighed into digestion vessels, followed by the addition of 2 mL of HNO_3_ and 2 mL of H_2_O_2_. Directional infrared heating was applied until complete digestion of the organic matrix was achieved. After digestion, the solutions were allowed to cool to room temperature and were quantitatively transferred and diluted to a final volume of 10 mL with ultrapure water.

Digestion efficiency was evaluated through residual acidity (RA), dissolved organic carbon (DOC), and residual solids (RS), providing a comprehensive assessment of matrix decomposition. The visual clarity of the solutions and the absence of residual particulate matter were also verified (Cora Jofre et al., 2020; 2021). All resulting solutions were directly suitable for ICP-MS analysis without additional treatment.

### 2.3 Instrumentation

Elemental determinations were carried out using an ICP-MS (ELAN DRC-e, PerkinElmer SCIEX, Thornhill, Canada). High-purity argon gas (minimum purity 99.996%) was used for plasma generation and was supplied by a local provider.

Sample introduction was performed using a high-performance, HF-resistant perfluoroalkoxy (PFA) nebulizer (model PFA-ST) coupled to a quartz cyclonic spray chamber equipped with an internal baffle and drain line. The spray chamber was temperature-controlled using a PC3 cooling system (ESI, Omaha, NE, USA) to ensure signal stability. A peristaltic pump fitted with black/black Tygon tubing (0.76 mm internal diameter, 40 cm length) was used for sample delivery. The monitored isotopes and instrumental operating conditions are summarized in Table 1. Isotopes used for MICal quantification are highlighted in bold, while those used for EC are underlined. Isotopes marked with both formats were used in both approaches.

**TABLE 1:** ICP-MS instrumental parameters and operating conditions employed for MICal.

Instrumental parameter	Operating condition
RF power (W)	1,050
Plasma gas flow rate (L min^-1^)	15
Auxiliary gas flow rate (L min^-1^)	4
Nebulizer gas flow rate (L min^-1^)	0.81
Sampler and skimmer cones	Nickel
Scanning mode	Peak hopping
Dwell time (ms)	50
Replicates number	3
Monitored isotopes	^**106**^ **Cd**, ^**108**^ **Cd**, ^**110**^ **Cd**, ^**111**^ **Cd**, ^112^Cd, ^113^Cd, ^**114**^ **Cd** , ^**116**^ **Cd**, ^**63**^ **Cu** , ^**65**^ **Cu**, ^92^Mo, ^**94**^ **Mo**, ^**95**^ **Mo** , ^**96**^ **Mo**, ^97^Mo, ^**98**^ **Mo**, ^**100**^ **Mo**, ^204^Pb, ^**206**^ **Pb**, ^**207**^ **Pb**, ^208^Pb, ^**50**^ **V**, ^**51**^ **V** , **^64^Zn**, **^66^Zn** , **^67^Zn**, **^68^Zn**, **^70^Zn**

### 2.4 Preparation of MICal solutions

For each digested sample, two calibration solutions were prepared following the MICal approach. Solution 1 (S1) consisted of a mixture of the sample digest and an analytical standard solution, while solution 2 (S2) was prepared by combining the same digest with the analytical blank, both at equal volumetric proportions (Figure 1). For each analyte, the concentration of the standard solution (C_std_) was selected individually based on preliminary tests, considering both the expected concentration range in the samples and the signal ratio obtained between S1 and S2. Following recommendations reported for MICal applications, C_std_ values were adjusted to achieve intermediate slope values whenever possible, to ensure robust calibration performance and minimize uncertainty in slope estimation (Cora Jofre et al., 2025). Standard concentrations were selected to ensure an adequate signal ratio between S1 and S2, thereby providing robust calibration performance. Elements were quantified within two analytical concentration ranges: ultra-trace range (0.5–10 μg L^-1^) for Cd, Pb, and V, and trace range (50–5,000 μg L^-1^) for Cu, Mo, and Zn.

### 2.5 MICal data treatment

Calibration curves were constructed by plotting the isotope signal intensity of the spiked solution (S1) against the corresponding intensity of the non-spiked solution (S2) for multiple naturally occurring isotopes. Linear regression was applied, and the analyte concentration (C_sample_) in the sample was calculated according to the MICal relationship (Equation 1):

$$C_{sample}=\frac{m\times C_{std}}{1-m}$$ (1)

where *m* is the slope of the MICal calibration plot and *C*_*std*_ is the concentration of the added standard.

Linearity (R^2^), slope consistency across isotopes, and the presence of outliers were evaluated for each element. Deviations from linearity or isolated points were interpreted as potential indicators of interferences.

### 2.6 External calibration (EC)

Conventional EC was performed for comparison purposes using the same digested samples and instrumental conditions employed for MICal analysis. Calibration curves were constructed using aqueous multielemental standard solutions prepared in an acid matrix similar to that of the sample digests. Three concentration levels were used to cover the expected analyte concentration range. The isotopes monitored for EC are indicated in Table 1 (underlined), to distinguish them from those evaluated but not selected for MICal.

### 2.7 Validation

Method validation was carried out following established criteria for ICP-MS analysis and previous MICal-based studies.

Linearity was evaluated through the MICal approach by evaluating the goodness of fit of the MICal plots for each analyte, ensuring adequate slope values and determination coefficients.

LOD and LOQ were estimated using an error propagation strategy. This calculation considers C_std_, the uncertainty associated with the calibration slope (expressed as the standard deviation of the slope–S_m_–), and the slope value itself (*m*). The procedure was implemented according to previously reported by Virgilio et al. (2020), and the corresponding expressions are presented in Equations 2, 3:

$$LOD=3*\left(\frac{C_{std}*S_{m}}{{\left(1-m\right)}^{2}}\right)$$ (2)

$$LOQ=10*\left(\frac{C_{std}*S_{m}}{{\left(1-m\right)}^{2}}\right)$$ (3)

Precision was evaluated in terms of repeatability by analyzing selected samples in triplicate (n = 3) under identical experimental conditions, considering both digestion and instrumental replicates. Results were expressed as relative standard deviation (RSD).

Accuracy was assessed by recovery studies using certified or reference values. Although the sample mass employed was lower than the minimum mass indicated in the CRM certificate, the material was used as a practical reference for methodological evaluation, and satisfactory analytical performance was obtained in terms of recoveries, precision, and calibration behavior.

The robustness of the calibration strategy was examined by monitoring slope stability and identifying potential deviations associated with isotope-specific spectral or matrix-related interferences, taking advantage of the intrinsic diagnostic capability of MICal previously reported for challenging sample compositions.

All measurements were performed in triplicate (n = 3), unless otherwise specified.

## 3 Results and discussion

### 3.1 MICal calibration behavior

**FIGURE 2:**
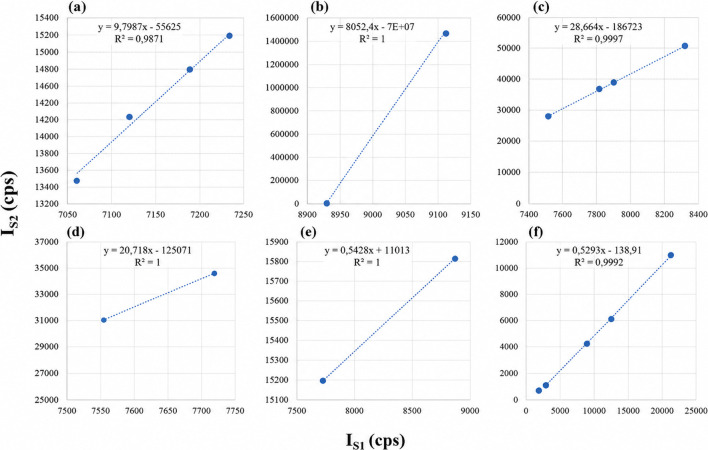
MICal calibration plots for **(a)** Cd, **(b)** Cu, **(c)** Mo, **(d)** Pb, **(e)** V, and **(f)** Zn obtained by ICP-MS analysis of bovine liver SRM.

The analytical performance of the proposed IRAD–MICal–ICP-MS workflow was first evaluated using a certified reference material (bovine liver). The MICal approach was assessed by constructing the calibration plots correlating the signal intensities of S2 (digest + blank) with those of S1 (digest + standard) for each monitored isotope. As shown in Figure 2, clear linear relationships were obtained for all selected analytes, with coefficients of determination (R^2^) higher than 0.9871, indicating that the MICal model adequately described the analytical response under the applied experimental conditions. The lowest R^2^ value was observed for Cd (0.9871), which may be associated with the analytical complexity of Cd determination by ICP-MS. This element presents some naturally abundant isotopes that may be affected by isobaric or polyatomic interferences, particularly from MoO^+^ species, and other matrix-dependent spectral contributions (Guo et al., 2010; Machado et al., 2017). Although MICal allows the identification and exclusion of clearly compromised isotopes, slight residual unresolved interferences may still contribute to signal dispersion. In addition, Cd was determined at ultra-trace concentration levels, where lower signal intensities increase susceptibility to instrumental noise and background fluctuations, could result in slightly higher variability in the S1 versus S2 relationship and consequently in a lower determination coefficient.

During this evaluation, certain isotopes were excluded from quantitative analysis (Table 1) due to the anomalous behavior observed in their MICal calibration plots. The isotopes finally selected for quantitative determination are explicitly identified in Table 1: those used for MICal quantification are highlighted in bold, while those used for EC are underlined. Isotopes marked with both formats were employed in both approaches. For elements such as Cd, selected isotopes were suitable for both MICal and EC. For elements strongly affected by spectral interferences, only the most reliable isotopic responses were retained for MICal quantification. This selection ensured that final concentrations were calculated using only isotopes showing consistent linearity, acceptable slope behavior, and absence of significant interference-related deviations.

Excluded isotopes typically exhibited deviations from linearity or inconsistent responses relative to the remaining isotopes of the same element, appearing as outliers in the S1 *versus* S2 signal plots. Specifically, isotopes were considered compromised when they showed (i) marked departures from the linear trend defined by the majority of isotopic responses, (ii) disproportionately high residuals relative to the fitted calibration line, or (iii) inconsistent slope values compared to the other isotopes of the same element. Such behavior is indicative of unresolved isotope-specific spectral interferences (including isobaric or polyatomic species contributing to the same m/z values) or matrix-related effects, affecting their analytical signals. The identification and exclusion of these compromised isotopes represent an intrinsic advantage of MICal, as previously reported (Virgilio et al., 2018).

For certain elements (Cu, Pb, and V), calibration was based on limited number of isotope points, in some cases only two. This situation arises either from the exclusion of affected isotopes due to spectral or matrix-related interferences, or from the intrinsic isotopic composition of the element, which may provide only a limited number of naturally abundant and analytically suitable isotopes. In the MICal approach, calibration is based on the direct relationship between paired signals obtained from S1 and S2, such that each valid isotope defines an independent calibration point and contributes to the construction of the calibration function, with a unique calibration slope. Consequently, even when only two non-interfered isotopes remain available, the resulting calibration remains mathematically well-defined and fully consistent with the conceptual basis of MICal.

Importantly, the exclusion of interfered isotopes did not compromise the analytical performance. On the contrary, restricting quantification to carefully selected isotopes resulted in robust calibration and consistent quantification across the studied matrices. This diagnostic capability, which is not available in EC or SA approaches, represents a distinctive strength of MICal when applied to complex samples.

Previous MICal studies have suggested that optimal performance is typically achieved when calibration slopes fall within an intermediate range (0.1 < slope <0.9) (Machado et al., 2018; Virgilio et al., 2018). In the present study, the calibration slopes obtained for V and Zn fell within this recommended interval, reflecting an appropriate selection of standard concentrations and balanced signal contributions from both calibration solutions. For other elements (Cd, Cu, Mo, and Pb), slope values outside this range were observed; however, these deviations occurred while maintaining acceptable linearity and well-defined calibration trends.

This behavior does not indicate a limitation of the MICal model but rather reflects element-specific sensitivity, differences in concentration ratios between S1 and S2, and instrumental response characteristics under the selected operating conditions. The validity of the calibration in these cases is further supported by the analytical figures of merit discussed in Section 3.2.

### 3.2 Analytical performance of MICal and comparison with EC

The analytical performance of the MICal approach was evaluated in terms of LOD, LOQ, accuracy, and precision, and compared with EC. As summarized in Table 2, MICal provided reliable quantification for all evaluated elements. The calibration curves for EC are presented in Supplementary Figure S1.

**TABLE 2:** Analytical parameters for MICal and EC employing ICP-MS.

Analyte	MICal					EC	
	LOD	LOQ	LOQ	Recovery	RSD	LOQ	Recovery
	(µg L^-1^)	(µg L^-1^)	(mg kg^-1^)	(%)	(%)	(mg kg^-1^)	(%)
Cd	0.97	3.2	0.22	106	5	0.28	3
Cu	0.43	1.4	0.09	99	0.02	0.24	70
Mo	0.07	0.25	0.02	101	1	0.29	89
Pb	1.8	5.9	0.39	102	0.08	0.41	94
V	0.18	0.61	0.04	85	3	1.8	n.c.^*^
Zn	0.21	0.70	47	111	0.2	0.36	112

* n.c. = not calibrated.

Recoveries obtained using MICal ranged from 85% to 111%, demonstrating satisfactory accuracy according to commonly accepted performance criteria for elemental analysis. Excellent recoveries were achieved for Cd, Cu, Mo, Pb, and Zn (99%–111%), while V showed a slightly lower recovery (85%), although still within acceptable limits for complex matrices. This lower recovery may be associated with the intrinsic analytical difficulty of V determination by ICP-MS. Unlike other analytes, V provides only two isotopes for MICal evaluation (Table 1). The isotope ^51^V may be affected by polyatomic species such as ^35^Cl^16^O^+^, ^14^N^16^O^21^H^+^, and ^34^S^16^O^1^H^+^, especially in agroalimentary matrices containing residual salts, nitrogen-containing compounds, and organic matter. These factors could increase the analytical difficulty of V determination and help explain the slightly lower recovery observed, while MICal still ensured reliable quantification through intrinsic matrix matching.

Precision was consistently high, with RSD below 5% for all elements, confirming the robustness and repeatability of the MICal approach.

The LOQ achieved by MICal (0.02–47 mg kg^-1^) were suitable for both nutritional (Cu, Mo, V, and Zn) and toxic trace elements (Cd and Pb). Notably, MICal allowed the determination of V at lower LOQ values than those achieved by EC, highlighting its improved sensitivity under the evaluated conditions.

The performance of EC provided acceptable recoveries for most elements (70%–112%). While, exhibited poorer accuracy for Cd and Cu. For V, EC failed to provide reliable results, yielding negative concentration values, which reflects its limited ability to compensate for matrix effects in complex samples. These limitations are well-known for EC when applied to complex matrices with high organic content or variable composition, where plasma-related and spectral interferences are not adequately compensated. MICal inherently overcomes these limitations by incorporating the sample matrix into both calibration solutions, ensuring effective matrix matching without the need for multiple spiking levels or individual calibration curves for each sample.

As discussed by Virgilio et al. (2020), multi-signal calibration approaches such as MICal may present higher LOD and LOQ compared to EC when estimated using error propagation. In the present study, LOQ values obtained by MICal were comparable or better than those obtained by EC for most analytes, except for Zn. This behavior may be attributed to the influence of standard concentration on slope uncertainty, as higher concentrations can increase variability in slope estimation and consequently affect detection limits. Notably, the LOD and LOQ obtained for Mo are consistent to those reported by Virgilio et al. (2020), supporting the validity of the calibration strategy.

Overall, the comparison with EC highlights that the improved analytical performance of MICal is closely related to its intrinsic matrix-matching capability. While EC provided acceptable results for some analytes, its performance was strongly influenced by matrix composition, leading to inconsistent recoveries and, in some cases, unreliable quantification. In contrast, by incorporating the sample matrix into both calibration solutions, MICal enables accurate and consistent quantification across heterogeneous samples, while also facilitating the identification of compromised isotope responses through calibration behavior.

Although SA is commonly used to overcome matrix effects in complex matrices, its implementation is considerably more laborious because each sample requires the preparation of several fortified solutions to construct individual calibration curves. In contrast, MICal simplifies this procedure by requiring only two solutions per sample, reducing reagent consumption, analysis time, and operational complexity, while still providing effective matrix compensation. This simplified workflow improves analytical throughput and makes MICal particularly attractive for routine multielemental analysis of complex matrices. The analytical advantages arising from its synergy with IRAD, as well as their implications from GAC and WAC perspectives, are discussed in the following sections.

### 3.3 Green analytical chemistry (GAC) assessment

Beyond analytical performance, the proposed IRAD-MICal-ICP-MS workflow was examined from a GAC perspective. The assessment considered both AGREE and AGREEprep frameworks, which are designed to evaluate the environmental impact of analytical methods and sample preparation steps, respectively (Supplementary Figure S2).

It should be noted that AGREE and AGREEprep are metric-based tools whose outputs depend on the predefined input parameters. In particular, AGREEprep is explicitly restricted to sample preparation variables, such as reagent type and volume, digestion time, and energy source, and does not consider calibration-related aspects (including the number of calibration solutions, standard consumption, and waste generation during calibration. Consequently, when identical digestion protocols are applied, AGREEprep is unable to discriminate between different calibration strategies, such as EC and MICal (Supplementary Figure S2), even though these approaches differ substantially in operational complexity, reagent use, and waste generation.

Similarly, AGREE metric provides a global assessment of analytical methods based on 12 GAC principles but does not explicitly treat calibration strategies as an independent stage of the analytical workflow. Therefore, the lack of differentiation between EC and MICal in AGREE or AGREEprep evaluations should be interpreted as a limitation of the scope of these tools, rather than as evidence of comparable environmental performance (Pena-Pereira et al., 2020; Pena-Pereira et al., 2022; Pena-Pereira et al., 2022b; Wojnowski et al., 2022).

From a sample preparation standpoint, IRAD offers several advantages aligned with GAC principles. The method significantly reduces acid consumption and digestion time compared with conventional microwave-assisted or block digestion procedures, while maintaining effective mineralization of agroalimentary matrices. Compared with conventional MWAD, IRAD requires lower reagent volumes (2 mL HNO_3_ and 2 mL H_2_O_2_), shorter digestion times (approximately 30 min for 18 samples), and lower energy demand due to the use of a 500 W infrared system, whereas MWAD typically involves higher acid volumes, longer heating cycles, and higher power input (approximately 1400 W). These features significantly reduce chemical hazards, waste generation, and overall energy consumption while maintaining suitable mineralization of agroalimentary matrices. Reduced reagent volumes translate into lower chemical hazards and decreased waste generation, while shorter heating cycles and lower energy requirements improve overall energy efficiency (Cora Jofre and Savio, 2024).

From a calibration perspective, the MICal strategy further enhances the greenness of the analytical workflow by requiring only two calibration solutions per sample, in contrast to EC or SA, which typically involve multiple standards and calibration points. This reduction in solution preparation lowers reagent consumption, minimizes analytical waste, and reduces operator handling.

Overall, the integration of IRAD and MICal results in a simplified, low-consumption, and energy-efficient analytical workflow that aligns well with the GAC principles, achieving sustainability improvements without compromising analytical accuracy, precision, or detection capability.

To address calibration-related aspects beyond the scope of AGREE and AGREEprep, the WAC framework was therefore applied, enabling a more comprehensive comparison of the entire analytical workflow, including calibration-related contributions to waste generation and operational efficiency.

### 3.4 White analytical chemistry (WAC) perspective

The analytical strategy developed in this study was evaluated according to the principles of WAC, which integrates analytical performance (red), environmental sustainability (green), and practical and socio-economic aspects (blue) into a single conceptual framework (Nowak et al., 2021). This approach enabled a comprehensive comparison between the IRAD-MICal-ICP-MS and IRAD-EC-ICP-MS workflows (Supplementary Table S1). Scores were assigned following the fit-for-purpose philosophy proposed by Nowak et al. (2021), considering multielemental ICP-MS analysis of complex agroalimentary matrices as the target application.

Although both strategies share identical digestion pretreatment and instrumental configuration, their overall performance profiles differed markedly. The MICal-based approach exhibited a substantially higher overall whiteness (85.3%) compared to EC (66.7%) (Supplementary Figure S3; Supplementary Table S1), mainly driven by improvements in analytical performance (red dimension) and operational efficiency (blue dimension). In particular, MICal demonstrated enhanced robustness in terms of accuracy, precision, and LOD, which is especially relevant when dealing with complex agri-food matrices prone to matrix effects and instrumental drift.

From an analytical performance (red) perspective, the IRAD-MICal-ICP-MS workflow meets established quality requirements for food analysis, as demonstrated by excellent linearity, adequate LODs and LOQs, good precision, and accurate recoveries (Section 3.2).

Regarding practicality and usability (blue dimension), the workflow provides a simplified analytical protocol with reduced calibration effort and sample preparation time, fewer handling steps, and lower operator workload. These features enable higher sample throughput and improved its suitability for routine laboratory implementation.

From an environmental (green) perspective, the main advantage of MICal arises the reduced number and volume of calibration solutions, leading to lower reagent consumption and decreased waste generation, rather than from changes in sample digestion conditions. This finding highlights that calibration strategies, often overlooked in greenness assessments, can significantly influence the overall environmental footprint of analytical workflows.

Importantly, the WAC assessment does not imply that MICal reduces the inherent complexity of ICP-MS instrumentation. Instead, it demonstrates that, within a high-performance analytical platform, MICal enables a more balanced compromise between analytical quality, environmental sustainability, and operational efficiency. Overall, this balanced performance supports the suitability of the IRAD-MICal-ICP-MS workflow as a holistic and forward-looking strategy for multielemental analysis in complex agroalimentary matrices.

### 3.5 Application to real agroalimentary samples

**FIGURE 3:**
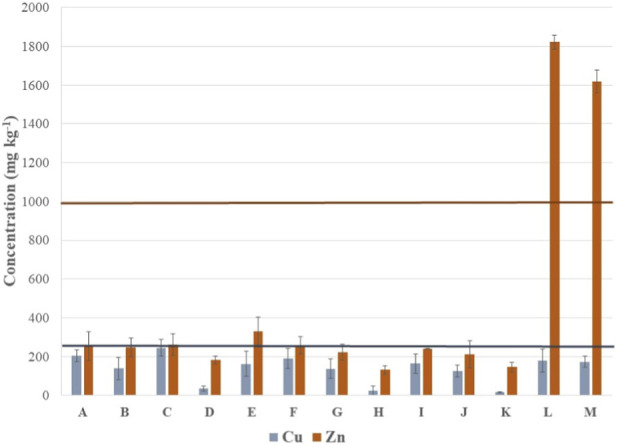
MICal-ICP-MS determination of Cu and Zn in compound feeds and agroalimentary samples.

**FIGURE 4:**
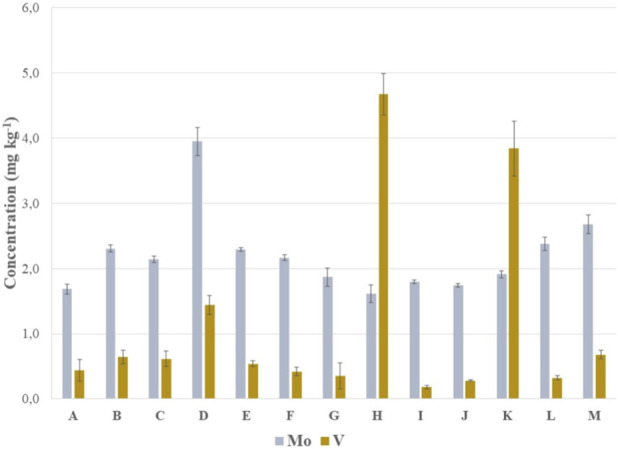
MICal-ICP-MS determination of Mo and V in agroalimentary samples.

The applicability of the proposed MICal-based methodology was demonstrated through the analysis of real agroalimentary samples, including compound feeds and animal-derived matrices. Elemental concentrations were determined using the MICal approach under the optimized experimental conditions described above, and the results are summarized in Figures 3, 4.

Cu and Zn concentrations were evaluated in the context of established dietary recommendations and tolerance limits for swine nutrition. Typical inclusion levels range from approximately 5–20 mg Cu kg^-1^ and 50–180 mg Zn kg^-1^ in growing and nursery pig diets, while maximum tolerable levels of 250 mg Cu kg^-1^ and 1,000 mg Zn kg^-1^ in complete feeds have been proposed to prevent adverse health effects (solid horizontal reference line in Figure 3) (National Research Council, 2005; National Research Council, 2012).

As shown in Figure 4, most samples exhibited Cu and Zn concentrations below these maximum tolerable limits, confirming their suitability for nutritional use. Samples L and M showed Zn concentrations exceeding 1,000 mg kg^-1^, as indicated by the solid horizontal reference line in Figure 3. Nevertheless, such elevated Zn levels are commonly associated with the intentional supplementation of zinc oxide in weanling pig diets, where concentrations of 2000–3,000 mg Zn kg^-1^ are temporarily employed to promote growth and control post-weaning diarrhea. These findings indicate that the proposed method is suitable not only for routine nutritional assessment but also for monitoring high-supplement formulations (National Research Council, 2005).

Mo and V, while biologically relevant, are not assigned specific dietary requirement values and are instead regulated based on tolerance considerations. The maximum tolerable levels reported for swine feed are approximately 150 mg Mo kg^-1^ and 10 mg V kg^-1^ (National Research Council, 2005; National Research Council, 2012). The concentrations determined in the analyzed samples were well below these tolerance thresholds (Figure 3), indicating no risk of excessive exposure and supporting the applicability of the method for monitoring elements that are relevant from a toxicological rather than nutritional standpoint (National Research Council, 2005).

Toxic elements such as Cd and Pb were either below the LODs or present at trace levels far below recommended maximum concentrations for swine feed (e.g., 10 mg kg^-1^ according to NRC guidelines) (National Research Council, 2005). These results indicate the absence of significant contamination in the analyzed agroalimentary samples and further support the suitability of the proposed methodology for food and feed safety assessment.

The consistent analytical performance observed across all samples type demonstrates the robustness of MICal approach for routine multielemental analysis. The method effectively handled heterogeneous compositions without the need for matrix-specific calibration strategies, providing a practical and efficient alternative for elemental determination in food and feed control laboratories.

## 4 Conclusion

This study demonstrates that MICal constitutes a robust, informative, and efficient calibration strategy for multielement analysis of complex agroalimentary matrices by ICP-MS. By incorporating the sample directly into the calibration process, MICal enables intrinsic matrix matching, ensuring accurate and precise quantification across samples with markedly different compositions, while offering diagnostic capabilities for identifying isotope-specific spectral and matrix-related interferences.

Compared with conventional EC, MICal exhibited superior robustness against matrix effects without the increased complexity and reduced analytical throughput typically associated with SA methods. Its implementation does not require additional instrumental modifications, and involves minimal calibration effort, supporting its straightforward application in routine food and feed analysis.

The performance of MICal is further strengthened when combined with IRAD. The clear digests obtained by IRAD are fully compatible with MICal calibration, ensuring consistent analytical performance without increasing procedural complexity. In addition, IRAD reduces acid consumption, digestion time, and energy demand compared with conventional digestion approaches, contributing to a more sustainable analytical workflow.

The benefits of this integration are effectively captured through the WAC framework, which highlights the balance achieved between analytical performance, environmental sustainability, and operational efficiency.

Overall, the IRAD–MICal approach represents a comprehensive and forward-looking strategy for multielement determination in agroalimentary matrices, with clear applicability in quality control, nutritional evaluation, and regulatory monitoring.

## Data Availability

The original contributions presented in the study are included in the article/Supplementary Material, further inquiries can be directed to the corresponding author.
