# Supplementary material for: Integrating multi-isotope calibration and infrared-assisted digestion for robust and sustainable multielemental determination in agroalimentary matrices by ICP-MS
Source: Front Chem. 2026 Jun 15;14:1799245. doi: 10.3389/fchem.2026.1799245 (PMC13310896; doi:10.3389/fchem.2026.1799245)
Supplement: Supplementary file 1 [file DataSheet1.docx]

Supplementary Material

# Supplementary Figures


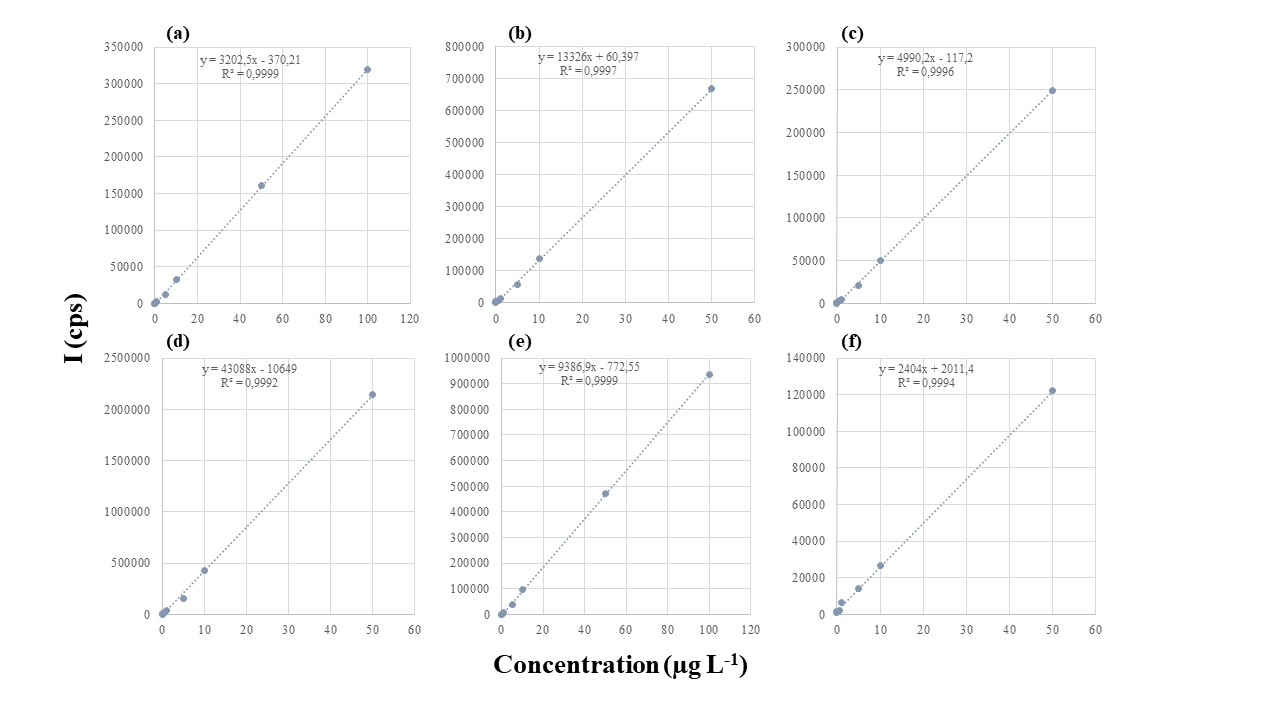


**Figure S1.** EC calibration plots for (a) ^114^Cd, (b) ^63^Cu, (c) ^95^Mo, (d) ^208^Pb, (e) ^51^V, and (f) ^66^Zn obtained by ICP-MS analysis.

**(a) (b)**


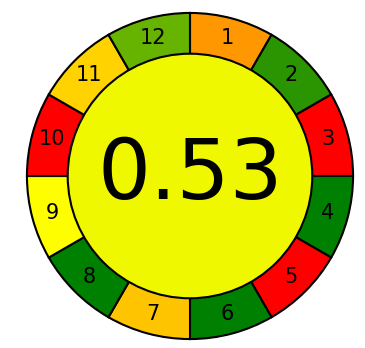

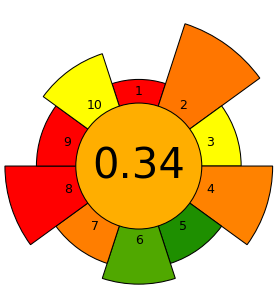


**Figure S2. (a)** AGREE and **(b)** AGREEprep assessment of IRAD-MICal-ICP-MS and IRAD-EC-ICP-MS analytical strategies highlighting similar scores arising from identical analytical methods and sample preparation step.

**(a) (b)**

**Figure S3.** WAC assessment comparing **(a)** IRAD-MICal-ICP-MS and **(b)** IRAD-EC-ICP-MS workflows.

# Supplementary Tables

**Table S1.** WAC assessment criteria, scores, and rationale used to compare IRAD-MICal-ICP-MS and IRAD-EC-ICP-MS approaches.

| **WAC Criterion** | **Description** | **IRAD–MICal–ICP-MS Score** | **Rationale (MICal)** | **IRAD–EC–ICP-MS Score** | **Rationale (EC)** |
| --- | --- | --- | --- | --- | --- |
| **R1 – Scope of application** | Applicability across matrices, analyte range, multi-element capability | **100** | Intrinsic matrix matching (sample present in both solutions) improves applicability across heterogeneous agroalimentary matrices | **80** | External standards may not match complex matrices; applicability reduced when matrix effects vary strongly |
| **R2 – LOD/LOQ** | Achievable detection/quantification limits under routine conditions | **80** | Improved robustness against matrix suppression/enhancement supports consistent low LOQ in complex samples | **50** | LOQ more sensitive to matrix mismatch and drift; poorer performance expected in variable matrices |
| **R3 – Precision** | Repeatability/reproducibility (e.g., RSD%, stability) | **100** | Two-solution design with shared matrix improves signal stability; reduced propagation of preparation variability | **80** | Precision impacted by matrix-dependent signal variability and calibration instability |
| **R4 – Accuracy** | Trueness vs CRMs / recoveries / bias | **100** | Recoveries consistent with CRMs; matrix-matched calibration reduces bias | **50** | Higher risk of bias from matrix mismatch; accuracy depends strongly on matching and control of interferences |
| **G1 – Toxicity of reagents** | Hazards/health and environmental toxicity of chemicals used | **80** | IRAD acids/oxidants | **80** | IRAD acids/oxidants |
| **G2 – Amount of reagents and waste** | Volume/mass of reagents; number of solutions; waste generation | **90** | Two calibration solutions; fewer standards prepared; reduced waste from calibration stage | **50** | Multiple standards/levels typically required; higher solution preparation and waste |
| **G3 – Energy and other media** | Energy demand, auxiliary media/consumables | **80** | IRAD digestion and ICP-MS operation | **80** | IRAD digestion and ICP-MS operation |
| **G4 – Direct impacts** | Emissions, wastewater generation, overall direct environmental burden | **93.3** | Comparable operational impacts; reduced calibration waste provides modest improvement | **80** | Comparable digestion/instrument impacts; calibration contributes more waste |
| **B1 – Cost efficiency** | Cost per analysis (consumables, standards, labor) | **100** | Lower labor and standard consumption; fewer solutions and preparation steps | **80** | More standards/preparation time increase overall cost |
| **B2 – Time efficiency** | Throughput; preparation and calibration time | **100** | Two-solution calibration reduces time and enables higher throughput | **80** | Longer calibration preparation; potential rework if matrix mismatch occurs |
| **B3 – Requirements** | Instrumentation, expertise, infrastructure needed | **100** | No additional instrumentation beyond ICP-MS; MICal uses routine isotopic signals | **80** | Similar ICP-MS requirements; more calibration handling and QC to maintain accuracy |
| **B4 – Operational simplicity** | Simplicity/portability/user-friendliness | **0** | ICP-MS workflows remain complex regardless of calibration approach | **0** | ICP-MS workflows remain complex regardless of calibration approach |

**
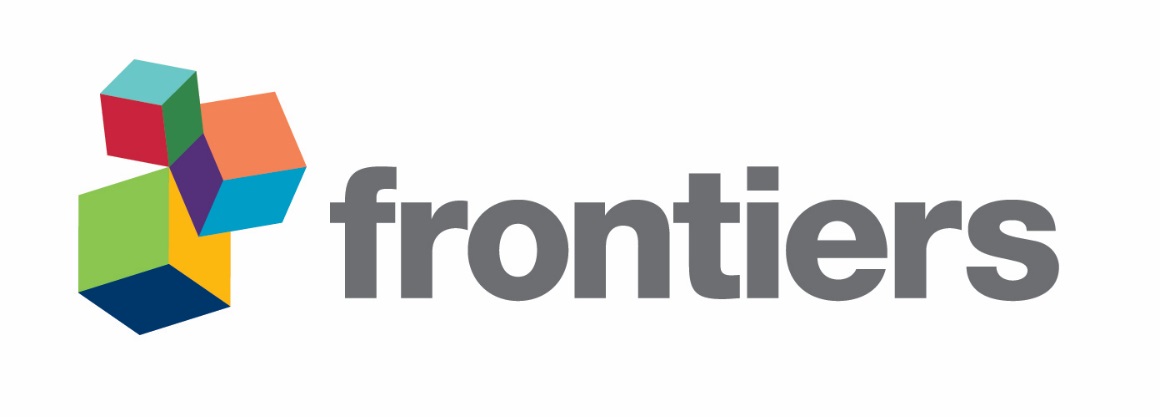
**
